# Case report: necrotizing fasciitis with ascending mediastinal emphysema secondary to anastomotic perforation after stapled hemorrhoidopexy

**DOI:** 10.3389/fmed.2026.1914139

**Published:** 2026-10-27

**Authors:** Ziwen Wang, Zhiyin Deng, Jingkai Wang, Haoyan Li, Shiwei Chen, Lisi Jian

**Affiliations:** 1 Department of Coloproctology, The Affiliated Guangzhou Hospital of TCM of Guangzhou University of Chinese Medicine, Guangzhou, China; 2 Department of Coloproctology, The Affiliated Traditional Chinese Medicine Hospital of Guangzhou Medical University, Guangzhou, China

**Keywords:** case report, mediastinal emphysema, necrotizing fasciitis, postoperative complications, stapled hemorrhoidopexy

## Abstract

**Introduction:**

Necrotizing fasciitis (NF) is a life-threatening soft-tissue infection that progresses rapidly, most commonly secondary to surgery or trauma. Stapled hemorrhoidopexy (Procedure for Prolapse and Hemorrhoids, PPH) is a minimally invasive procedure for mixed hemorrhoids; however, anastomotic leakage resulting from a staple-line defect and plausibly related to staple tension or impaired local perfusion can rarely induce enterogenic NF, with ascending mediastinal emphysema being uncommon as a co-presentation with surgically confirmed NF.

**Case presentation:**

A 37-year-old previously healthy male developed severe perianal pain and a high fever (39.3 °C) on postoperative day 2 after PPH, followed by subcutaneous crepitus with clinical deterioration on postoperative day 3, which prompted transfer to our hospital. CT showed widespread gas spreading from the perianal and ischiorectal fossae through the pelvic and retroperitoneal fascia to the posterior mediastinum and cervical fascial spaces, demonstrating gas tracking along necrotizing fasciitis of the perianal, perineal, ischiorectal, pelvic, and retroperitoneal compartments—subsequently confirmed at surgery—together with radiologically demonstrated ascending mediastinal emphysema; no mediastinal collection or abscess was seen. Emergency transverse colostomy, extensive fascial debridement, broad-spectrum antibiotic therapy, and supportive care were performed. The patient recovered fully, with no recurrence at the 1-year follow-up.

**Discussion:**

This case highlights the unique fascial plane of spread of the infection and demonstrates a continuous anatomic progression pathway as its core finding: a small posterior post-PPH staple-line defect localized by digital rectal examination and directly measured at surgery, which progresses to rapidly progressive, surgically confirmed perineal, ischiorectal, and retroperitoneal necrotizing fasciitis, with extensive cephalad gas tracking into the posterior mediastinum and cervical spaces (no mediastinal collection) identified on CT. Early CT is pivotal for diagnosing extensive involvement, whereas prompt surgical source control and multidisciplinary collaboration are central to the favorable outcome. The multimodal regimen applied in this case aligns with established principles for necrotizing soft-tissue infections, and the independent contribution of any single intervention cannot be definitively concluded.

**Conclusion:**

Post-PPH patients with disproportionate pain or crepitus require high clinical suspicion of NF and urgent CT. The present observation adds to the documented anatomic pattern of cephalad gas tracking and underscores the need to distinguish radiologic gas tracking from surgically confirmed necrotizing infection; it does not establish that any particular intervention altered survival.

## 1 Introduction

Necrotizing fasciitis (NF) is an acute, life-threatening soft-tissue infection. It is characterized by rapidly progressive necrosis of the fascia and the subcutaneous tissue. It typically has a rapid onset and aggressive progression, posing diagnostic challenges and leading to high morbidity and mortality (1). This condition is most often secondary to trauma, surgical intervention, or an immunocompromised state. The fundamental pathogenesis involves bacterial invasion of the skin and subcutaneous tissue, which spreads swiftly along the superficial and deep fascial planes as well as through potential soft tissue spaces (2). In NF, the commonly affected sites include the perianal region, perineum, and extremities. If not recognized and treated promptly, NF can progress to septicemia, multi-organ failure, and death (3). Previous reports have indicated an incidence ranging from 3 to 50 cases per million population annually, with a mortality rate of 17%–35%. Radical surgical debridement remains an essential component of effective treatment (4).

Stapled hemorrhoidopexy (PPH) is a minimally invasive procedure for mixed hemorrhoids that has gained wide acceptance because of its reduced postoperative pain and short recovery time (5). However, as the number of treated cases has increased, various complications—such as anastomotic stenosis, rectal narrowing, and postoperative bleeding—have been reported, leading to a gradual decline in its clinical use (6). In extremely rare circumstances, disruption or ischemia at the staple line may cause leakage of intestinal bacteria, resulting in deep-tissue infection and secondary necrotizing fasciitis. Cephalad gas tracking along fascial spaces with mediastinal emphysema has been reported as a benign extraperitoneal air leak through the staple line (7, 8); however, its coexistence with surgically confirmed necrotizing fasciitis extending from the perineum and retroperitoneum is less well characterized.

This report describes a case of NF following stapled hemorrhoidopexy complicated by anastomotic leakage, in which a small posterior staple-line defect was localized preoperatively by digital rectal examination and was directly visualized and measured at surgery; intraoperative exploration confirmed necrotizing fasciitis involving the perianal, perineal, ischiorectal, pelvic, and retroperitoneal compartments, while CT demonstrated continuous cephalad gas tracking into the posterior mediastinum and cervical spaces without mediastinal collection or abscess. We additionally performed a focused, reproducible literature search (databases, date, and terms detailed in the Discussion) and compared the present case with the most closely related published reports (Supplementary Table S1). Based on the patient's clinical course, imaging findings, and management outcomes, together with relevant domestic and international literature, this paper discusses the pathogenesis, diagnostic approach, and therapeutic strategies for this rare postoperative infection, underscoring the importance of timely recognition, radiologic evaluation, and early surgical intervention. This work has been reported in line with the SCARE Guidelines 2025 (9) and is compliant with the TITAN Guidelines 2025 (10).

## 2 Case presentation

A previously healthy 37-year-old male underwent PPH for mixed hemorrhoids at an outside institution. The surgery was uneventful. Two days after surgery, the patient complained of acute anal pain and a high fever of 39.3 °C. Although antipyretics provided transient relief, the pain worsened significantly. It radiated upwards to the lower abdomen and was accompanied by shortness of breath. On the third postoperative day, his temperature increased again to 38.4 °C. The patient was transferred to our hospital for further management. Physical examination revealed extensive swelling and erythema of the perianal and gluteal regions, with palpable crepitus. Digital rectal examination revealed deep tenderness at the posterior rectal wall with a depression at the staple line. The pain was much greater than that typically expected following routine PPH. Laboratory results showed a white blood cell count (WBC) of 17.98 × 10⁹/L, neutrophil percentage 89.5%, C-reactive protein (CRP) 102.1 mg/L, and Serum Amyloid A (SAA) > 200.00 mg/L. Hemoglobin was 129 g/L, platelet count 200 × 10⁹/L, serum creatinine 71.6 μmol/L, potassium ion 3.3 mmol/L, liver function, and remaining electrolytes were within normal limits. Urgent contrast-enhanced CT of the pelvis, abdomen, and chest revealed widespread gas tracking from the perianal region and ischiorectal fossae through the pelvic and retroperitoneal fascial layers along the psoas and iliac muscles, extending superiorly through the diaphragmatic hiatus into the posterior mediastinum and even the cervical fascial spaces (Supplementary Figures S1A–G). No mediastinal fluid collection or abscess was observed. These imaging findings, together with the operative findings described below, confirmed the diagnosis of NF involving the perianal, perineal, ischiorectal, pelvic, and retroperitoneal compartments, accompanied by radiologically demonstrated ascending mediastinal emphysema. Mediastinal and cervical gas represented emphysematous tracking rather than surgically or microbiologically confirmed necrotizing mediastinitis. Of note, the approximately 0.5-cm posterior staple-line defect was directly visualized on CT as a small posterior defect approximately 3 cm above the dentate line; its precise extent and margins were further inferred radiologically, localized preoperatively by digital rectal examination (deep posterior wall tenderness with a palpable staple-line depression), and subsequently confirmed and roughly measured by gentle probing during surgical exploration. Radiologic findings, digital examination, and operative confirmation are reported separately throughout this manuscript.

On urgent surgical exploration (within 12 h of diagnosis), the stapled rectal anastomosis showed a full-thickness 0.5-cm defect on the posterior rectal wall, located approximately 3 cm above the dentate line, with active leakage of purulent fluid, confirming anastomotic leakage through a staple-line perforation. No significant loosening, shedding, cutting into the intestinal wall, or exposure of the anastomotic staples was observed intraoperatively at the defect site. Although severe edema and abundant necrotic purulent tissue precluded complete visualization of the entire staple line (as detailed in the Discussion), the focal defect itself had been localized preoperatively by digital rectal examination—which demonstrated a depression at the staple line with focal tenderness of the posterior wall—and could be directly visualized, gently probed, and measured intraoperatively before debridement; active leakage of purulent fluid through the defect established its full-thickness nature. Meanwhile, intraoperative exploration confirmed necrotizing fasciitis involving the perianal subcutaneous tissue, superficial perineal fascia, deep perineal fascia, bilateral ischiorectal fossae, and retroperitoneal space, accompanied by extensive fascial necrosis and foul-smelling pus. The cornerstone of the treatment strategy is radical debridement, with the primary goals of infection source control and saving the patient's life, the surgical procedure was performed in a sequential manner. First, a transverse double-barrel colostomy was constructed to achieve complete fecal diversion and eliminate ongoing enteric bacterial contamination. Subsequently, the anastomotic defect was evaluated: given the acute infection phase, severe tissue edema and extremely high risk of repair failure, neither primary repair of the defect nor staple removal was performed at this stage, in accordance with damage-control principles that prioritize source control and defer definitive reconstruction in the setting of severe contamination and physiological instability (11), and definitive management was planned after complete resolution of infection. Then, radical layered fascial debridement was carried out via a curved incision made 5 cm lateral to the anal verge at the 6 o'clock position. All necrotic fascia and non-viable tissue in the perianal region, perineum, pelvic cavity and retroperitoneal space were completely excised, and all involved infected cavities were thoroughly irrigated with hydrogen peroxide and povidone-iodine solution. Finally, double-lumen drainage catheters were placed transanally across the anastomotic defect, as well as in the bilateral ischiorectal fossae and retrorectal space for continuous postoperative normal saline irrigation and drainage. Meanwhile, rubber seton loops were placed between the left inguinal and left scrotal incisions to achieve dependent counter-drainage.

Postoperatively, the patient was admitted to the intensive care unit (ICU) for comprehensive management. Initial antibiotic therapy combined imipenem-cilastatin with metronidazole to cover both aerobic and anaerobic pathogens. Supportive treatment included fluid resuscitation, correction of metabolic acidosis, maintenance of adequate diuresis, and supplementation with albumin and electrolytes. Inflammatory markers and renal and hepatic function were monitored daily. To improve local oxygenation, the wound cavity was irrigated with metronidazole-saline solution, and the dressings were regularly changed to promote granulation tissue growth.

On postoperative day 5 (counted from the emergency surgery at our hospital), the patient's white blood cell count had decreased to 8.06 × 10⁹/L, returning to the normal reference range, and the C-reactive protein level had dropped to 24.2 mg/L, representing a reduction of more than 70% from baseline. In addition, the patient had remained afebrile and hemodynamically stable for over 72 consecutive hours. Intraoperatively, pus specimens were collected from all involved fascial spaces for aerobic, anaerobic, and fungal cultures, as well as antimicrobial susceptibility testing. All subsequent microbiological cultures returned negative for bacterial or fungal growth at 5 days of incubation; because no organism grew, formal susceptibility testing could not be performed. These negative results must be interpreted cautiously: specimens were obtained after broad-spectrum antibiotics had been initiated, and fastidious anaerobes may fail to grow despite appropriate specimen transport, so negative cultures do not prove sterility. The antimicrobial regimen was de-escalated to latamoxef monotherapy, an oxacephem with documented activity against Enterobacterales and many anaerobes (12), as an individualized step-down strategy; because no causative organism was isolated, microbiological confirmation of the regimen's adequacy was not possible, and the patient was monitored closely with a low threshold for re-escalation, to reduce the risk of antimicrobial resistance and minimize adverse drug reactions. Repeat CT on postoperative day 8 showed complete absorption of mediastinal gas and no further spread of infection (Supplementary Figure S1H). The patient was discharged on postoperative day 19, with satisfactory wound healing and stable colostomy function. Follow-up surgeries included high fistula repair with seton drainage and removal of the exposed staples at three months, V-Y flap reconstruction of the perianal defect at six months, and colostomy reversal at nine months. At one-year follow-up, anal function and perineal appearance were normal, and there was no recurrence or stenosis.

## 3 Discussion

The principal observation in this case is a documented anatomic chain: a small posterior staple-line defect (visualized on CT, localized by digital rectal examination, and directly measured at surgery), surgically confirmed perineal/ischiorectal/retroperitoneal necrotizing fasciitis, and radiographically continuous cephalad gas tracking into the posterior mediastinum and cervical spaces without mediastinal collection or abscess. A plausible mechanism is staple tension combined with relatively insufficient local blood supply in the anastomotic area, which may have allowed pathogenic intestinal flora to invade the fascial layer; however, no direct evidence of excessive staple tension or local ischemia was documented, and the precise mechanism of the defect remains unconfirmed. Such infections progress rapidly and are accompanied by significant systemic toxicity. Intraoperative exploration during the first surgery confirmed that the necrotizing fasciitis involved the perianal subcutaneous tissue, superficial and deep perineal fascia, and retroperitoneal space. Preoperative CT revealed that gas not only spread continuously in a cephalad direction along the ischiorectal fossae, pelvic cavity, and retroperitoneal fascia to the mediastinal fascial spaces and cervical fascial spaces, but also extended caudally and laterally to multiple intermuscular spaces including those of the psoas major muscle, presenting characteristic imaging findings of multidirectional spread (Supplementary Figures S1A–G). Of note, gas within the mediastinal and cervical compartments represented radiologically demonstrated cephalad tracking; necrotizing infection in these compartments was neither surgically nor microbiologically confirmed, and no mediastinal collection or abscess was identified. Compared to general postoperative subcutaneous emphysema, NF presents with severe pain and systemic signs of infection. CT is a key tool for early diagnosis and enables clinicians to promptly identify life-threatening complications. The literature confirms that patients who underwent surgery within 24 h of admission had a significantly higher survival rate than those who underwent surgery after 24 h. Early surgery (within 6 h) can further improve survival (13). Cirocco (14) reported 9 perforations, 6 cases of sepsis, and 2 cases of Fournier gangrene, with 3 deaths (POD 4, 6, and 10), underscoring the life-threatening potential of post-PPH deep sepsis and reinforcing the need for urgent source control. In this case, CT examination was performed immediately after the onset of symptoms, and surgical intervention was implemented within 24 h of diagnosis, an approach consistent with evidence that prompt source control is associated with improved outcomes in necrotizing soft-tissue infection (11, 13). Nevertheless, the contribution of this timing to survival cannot be isolated in a single uncontrolled observation.

The core of the treatment strategy is thorough debridement. In this case, emergency double-barrel transverse colostomy was performed to prevent distal intestinal contamination from reaching the wound site, and extensive debridement and drainage were performed for multiple fascial spaces, including the perianal region, pelvic cavity, and inguinal region, with a large amount of pus drained during the operation. During the first intraoperative exploration of the stapled anastomosis, a focal 0.5 cm defect on the posterior wall of the anastomosis was confirmed, approximately 3 cm above the dentate line; the defect was directly visualized and measured, and active purulent leakage through it established a full-thickness perforation, while no obvious staple loosening, shedding, intestinal wall cutting, or exposure was observed at the defect site. Severe intraoperative infection, marked tissue edema, and abundant necrotic and purulent tissue obscured the remainder of the surgical field and, together with the small size of the staples, precluded complete evaluation of the entire anastomotic staple line. Direct measurement of the focal defect—which had been localized preoperatively by digital rectal examination—is therefore not contradictory to the incomplete survey of the staple line as a whole. Concurrent exploration further verified the involvement of necrotizing fasciitis in the perianal subcutaneous tissue, superficial perineal fascia, deep perineal fascia, bilateral ischiorectal fossae, and retroperitoneal space, accompanied by extensive fascial necrosis and foul-smelling pus. Therefore, the primary goals of this surgical protocol were to control the source of infection and save the patient's life. First, a transverse double-barrel colostomy was performed to achieve fecal diversion and eliminate the source of intestinal bacterial contamination. Subsequently, radical debridement was conducted to resect all necrotic fascia and devitalized tissue in the perianal, perineal, pelvic, and retroperitoneal spaces, accompanied by thorough irrigation of all involved infected cavities. For the anastomotic defect identified intraoperatively, neither primary repair of the defect nor staple removal was performed during this operation. This decision was made in consideration of the patient's severe tissue edema and active infection, which would result in an extremely high failure rate of anastomotic repair at this stage; by contrast, fecal diversion combined with adequate drainage could promote spontaneous healing of the anastomotic defect. This damage-control strategy—achieving source control, diversion, and drainage in the contaminated, edematous field while deferring definitive repair—accords with contemporary source-control guidelines for emergency general surgery (11); the defect and exposed staples were definitively managed after resolution of infection during the staged operation at 3 months. Accordingly, only thorough debridement of the perianastomotic region was completed in this operation. Double-lumen drainage tubes were placed transanally across the anastomosis, in the bilateral ischiorectal fossae and the retrorectal space for continuous postoperative irrigation and drainage. Meanwhile, rubber seton loops were placed for counter-drainage between the left inguinal and left scrotal incisions.

The initial empiric intravenous antibiotic regimen consisted of imipenem-cilastatin combined with metronidazole, initiated immediately after intraoperative collection of pus for aerobic, anaerobic, and fungal cultures (with susceptibility testing requested), a regimen that provides broad-spectrum coverage against the common pathogens associated with perianal enterogenic necrotizing fasciitis, consistent with IDSA recommendations for broad empiric therapy in necrotizing soft-tissue infection, which must cover both polymicrobial aerobic–anaerobic infection and monomicrobial pathogens such as group A Streptococcus or MRSA (15). Following 5 days of empiric therapy, the antibiotic regimen was de-escalated to latamoxef, in the context of adequate surgical source control and documented clinical improvement—apyrexia since the first postoperative day, more than 72 h of hemodynamic stability, and a fall in WBC to 8.06 × 10⁹/L and CRP to 24.2 mg/L by postoperative day 5—together with negative aerobic, anaerobic, and fungal cultures at 5 days. Latamoxef is an oxacephem with documented *in-vitro* activity against Enterobacterales and many anaerobes, including Bacteroides fragilis (12), matching the anticipated enteric Gram-negative and anaerobic spectrum of this perianal infection; however, it does not provide universal coverage of all potential NSTI pathogens, notably MRSA and group A streptococci (15). Because no causative organism was isolated, negative cultures may reflect prior broad-spectrum antibiotic exposure or suboptimal recovery of fastidious anaerobes—the adequacy of the step-down regimen against the pathogen(s) responsible could not be microbiologically confirmed. The decision therefore rested on explicit clinical criteria: (i) adequate source control with no residual collection; (ii) hemodynamic stability and apyrexia for more than 72 h; (iii) WBC normalization and a greater-than-70% reduction in CRP; and (iv) negative aerobic, anaerobic, and fungal cultures at 5 days, with daily monitoring and a low threshold for re-escalation. This de-escalation strategy was adopted to narrow the antimicrobial spectrum, reduce the selective pressure exerted by broad-spectrum antibiotics on drug-resistant bacteria, and mitigate the risk of adverse events such as Clostridioides difficile infection linked to prolonged carbapenem administration. For wound management, double lumen drainage catheters were placed in the bilateral ischiorectal fossae and the retrorectal space (together with a transanal catheter across the defect) for continuous postoperative normal saline irrigation and drainage, and rubber seton loops were placed between the left inguinal and left scrotal incisions to achieve dependent counter-drainage; the infected cavities were irrigated with metronidazole-saline solution and the dressings were changed regularly to promote granulation. After the above comprehensive treatments, the patient's body temperature returned to normal on the first postoperative day, and a re-examination CT, 1 week later, showed complete absorption of mediastinal gas and complete resolution of the mediastinal emphysema, consistent with control of the infection pathway in this patient. The favorable outcome was temporally associated with prompt source control, extensive debridement, fecal diversion, drainage, antimicrobial therapy, and multidisciplinary supportive care, consistent with established principles for the management of necrotizing soft-tissue infection; the independent contribution of each component cannot be established from a single case.

Multiple studies have reported the transmission mechanism of necrotizing fasciitis. Roje et al. suggested that in cases originating from the genital region, infectious bacteria may spread through Buck's fascia of the penis. The infection can then extend along the Dartos' fascia of the scrotum and penis, Colles' fascia of the perineum, and Scarpa's fascia of the lower abdominal wall (16). Sakhri et al. reported a case of intra-abdominal infection caused by a rectal fistula, which invaded soft tissues primarily along the psoas major muscle or through pelvic foramina, such as the inguinal canal and obturator foramen, by contiguous spread, extending from the abdominal wall to the extremities. The involvement of the deep fascia of the extremities progresses rapidly, leading to necrosis of the adjacent tissues (17).

CT images in this case clearly showed that the infection originated in the perianal region and ischiorectal fossae. Gas then spreads upward to the mediastinum and cervical spaces via the pelvic and retroperitoneal fasciae. At the same time, it extended downward and outward to the bilateral psoas major, pectineus, sartorius, and gracilis muscle spaces, presenting characteristic imaging features of multidirectional spread (Supplementary Figures S1A–G). This dissemination pathway is consistent with the known principles of anatomical continuity.

 Gas and infectious agents can enter the retroperitoneal space via the extraperitoneal pelvic space. They then spread upward into the mediastinum through weak areas or natural foramina of the diaphragm and further extend to the deep cervical spaces via channels, such as the visceral and retropharyngeal spaces of the neck. Meanwhile, the infection can also spread downward and outward along the extraperitoneal space, extend to the scrotum through the inguinal canal, and spread to the thigh along the fascial sheath of muscles, such as the iliopsoas muscle. This series of anatomical channels constitutes a low-resistance anatomical space, enabling local infection to cause life-threatening extensive fascial necrosis and sepsis within a short time (18), as illustrated in the dedicated anatomical flow chart adapted from Frias Vilaça et al. (Supplementary Figure S2).

Therefore, imaging signs of continuous gas distribution should serve as a warning for the early detection of such catastrophic complications. For patients after anorectal surgery such as PPH, high clinical suspicion of NF should be maintained in the presence of disproportionate pain, fever, or subcutaneous crepitus, and urgent CT should be obtained to define the proximal and distal extent of gas tracking. Intraoperative exploration should clarify the exact anatomical scope and fascial layers of infection to guide radical layered debridement. For the management of anastomotic defects and staple lines in the acute infection stage, the principles of damage control surgery should be followed, with the core goal of infection source control and life preservation, and empiric anti-infective therapy should be formulated and adjusted based on the patient's clinical response, inflammatory marker trends, and the common pathogenic spectrum of the disease. Recognizing this sign should prompt urgent CT and surgical consultation.

Literature search and comparison with previously reported cases. A focused literature search was performed on 15 September 2026 in PubMed/MEDLINE, Embase, and Scopus. The search terms, combined with Boolean operators, were: (((((((((((((((((((perforation[Title/Abstract]) OR (dehiscence[Title/Abstract])) OR (anastomotic leak[Title/Abstract])) OR (pelvic sepsis[Title/Abstract])) OR (perineal sepsis[Title/Abstract])) OR (Fournier gangrene[Title/Abstract])) OR (necrotizing fasciitis[Title/Abstract])) OR (retroperitoneal[Title/Abstract])) OR (pneumoretroperitoneum[Title/Abstract])) OR (pneumomediastinum[Title/Abstract])) OR (mediastinal emphysema[Title/Abstract])) OR (cervical emphysema[Title/Abstract])) OR (subcutaneous emphysema[Title/Abstract])) OR (Surgical Wound Dehiscence[MeSH Major Topic])) OR (Perforation[MeSH Major Topic])) OR (Fournier Gangrene[MeSH Major Topic])) OR (Necrotizing Fasciitis[MeSH Major Topic])) OR (Pneumomediastinum[MeSH Major Topic])) OR (Subcutaneous Emphysema[MeSH Major Topic])) AND ((((((stapled hemorrhoidopexy[Title/Abstract]) OR (procedure for prolapse[Title/Abstract] AND hemorrhoids[Title/Abstract])) OR (stapled hemorrhoidectomy[Title/Abstract])) OR (Longo[Title/Abstract])) OR (Hemorrhoidectomy[MeSH Major Topic])) OR (Surgical Staplers[MeSH Major Topic])). Titles and abstracts were screened independently by two authors, and reference lists of included articles and relevant reviews were hand-searched. Among 19 full-text articles retrieved, we retained those directly comparable to the present observation across the dimensions requested above; large complication-series background data, bleeding/hematoma without infection, rectal obstruction/stricture without sepsis, long-term follow-up series, and letters commenting on other cases were cited only as background and are not rows in Supplementary Table S1. The retained reports are summarized in Supplementary Table S1. No prior report in our material documented, in one patient, all three of: (i) a DRE-localized and directly measured posterior staple-line defect; (ii) surgically confirmed perineal/ischiorectal/retroperitoneal NF; and (iii) continuous cephalad gas tracking into the posterior mediastinum and cervical spaces without mediastinal collection.

Incremental contribution and hypothesis generated. Disproportionate postoperative pain as a warning sign, the diagnostic importance of CT, early surgical source control, radical debridement, fecal diversion when appropriate, drainage, and broad-spectrum antimicrobial treatment are established principles of necrotizing soft-tissue infection (NSTI) care and are not presented here as the principal novelty of this report. Against this background, the incremental value of the present observation is the documentation, in a single patient, of a complete anatomic chain: (i) a small posterior staple-line defect visualized directly on CT, localized by digital rectal examination, and directly measured at surgery; (ii) the proximal necrotizing process surgically confirmed to involve the perineal, ischiorectal, and retroperitoneal fascial compartments, extending beyond the scrotal/inguinal subcutaneous necrosis described by Pessaux (19) and the presacral/retroperitoneal collection described by van Wensen (20); and (iii) the distal mediastinal and cervical findings representing gas tracking only, with no mediastinal collection or abscess, and therefore not treated as necrotizing mediastinitis, in contrast to the benign-barotrauma reports (7, 8) in which no necrotizing infection was present at all. This anatomic documentation, combined with the Supplementary Table S1 comparison, supports two testable hypotheses that prior reports do not jointly address. Hypothesis 1 concerns a damage-control strategy in the active-NF period: in this patient, primary repair of the staple-line defect was not attempted and the staples were not removed at the index operation, in the presence of NF with severe tissue edema; fecal diversion with wide drainage was relied upon, and the defect and exposed staples were managed in a staged fashion 3 months later, after infection control. This differs from Ryu and Bae (21), who performed primary laparoscopic closure of a free rectal perforation without NF, and from Pessaux et al. (19), who performed end-colostomy. The hypothesis is that, in active NF with severely edematous tissues, primary repair carries a high failure rate, and fecal diversion with drainage allowing spontaneous closure of the defect, followed by staged management, is a reproducible strategy. Hypothesis 2 concerns above-diaphragm gas tracking: CT demonstrated mediastinal and cervical gas tracking without mediastinal collection, pleural effusion, or referable hemodynamic deterioration, and no thoracic exploration was performed. The hypothesis is that, in PPH-associated NF, CT demonstration of mediastinal or cervical gas tracking without mediastinal collection should not automatically trigger thoracic exploration; the primary surgical target remains the perineal and retroperitoneal source, with thoracic management reserved for clinical or radiologic evidence of true mediastinitis (e.g., mediastinal fluid, pleural effusion, hemodynamic deterioration referable to the chest). Both hypotheses require testing in larger, preferably multi-institutional series; the present *N* = 1 observation is not offered as proof of either.

## 4 Limitations

This report has several limitations. First, it is a single-patient observation and cannot establish incidence, causality, or the superiority of any individual treatment component. Second, the precise mechanism of the staple-line defect—for example stapler misfiring, excessive staple tension, or delayed pressure ischemia—could not be established, because no direct evidence of ischemia or abnormal staple tension was documented. Third, no causative microorganism was isolated, most likely because specimens were obtained after initiation of broad-spectrum antibiotics; this precluded susceptibility-guided therapy and microbiological confirmation of the de-escalation regimen. Fourth, mediastinal and cervical involvement was demonstrated radiologically as gas tracking only and was not confirmed surgically or microbiologically as necrotizing infection (necrotizing mediastinitis); no mediastinal collection or abscess was identified. Fifth, the independent contributions of fecal diversion, non-repair of the staple-line defect, the drainage strategy, antimicrobial selection, and staged reconstruction cannot be separated in this case. Finally, inflammatory markers were measured at multiple time points as listed in Supplementary Table S2 (from outside-hospital transfer through the one-year follow-up), but were not sampled at every bedside reassessment, and procalcitonin was not measured, limiting the granularity of the documented response trajectory.

## 5 Conclusion

Anastomotic leakage after stapled hemorrhoidopexy rarely causes necrotizing fasciitis, which can spread along contiguous fascial planes and lead to ascending mediastinal emphysema; in this patient, mediastinal and cervical involvement was demonstrated radiologically as gas tracking and was not surgically confirmed necrotizing mediastinitis. Early CT imaging is essential for identifying these pathways and assessing the extent of the disease. Timely surgical source control, fecal diversion, and adjunctive multidisciplinary treatment were temporally associated with the favourable outcomes in this patient and are presented as established necrotizing soft-tissue infection principles, not as the novelty of this report. What this case adds, beyond these established principles, is a documented anatomic chain: a posterior staple-line defect visualized on CT, localized by digital rectal examination, and directly measured at surgery; surgically confirmed perineal/ischiorectal/retroperitoneal necrotizing fasciitis; and CT-demonstrated cephalad gas tracking into the posterior mediastinal and cervical spaces without mediastinal collection. This documentation supports two testable hypotheses. First, in active necrotizing fasciitis with severely edematous tissues, primary repair of the staple-line defect carries a high failure rate; fecal diversion with wide drainage, allowing spontaneous closure of the defect and staged management after infection control, appears to be a reproducible strategy, in contrast to primary closure performed in the absence of necrotizing infection (21) and to end-colostomy (19). Second, CT demonstration of mediastinal or cervical gas tracking without mediastinal collection should not by itself trigger thoracic exploration; the primary surgical target remains the perineal and retroperitoneal source, with thoracic management reserved for clinical or radiologic evidence of true mediastinitis. Both hypotheses require testing in larger, preferably multi-institutional series; the present *N* = 1 observation is not offered as proof of either. Recognizing disproportionate postoperative pain, persistent fever, or subcutaneous crepitus after anorectal surgery should prompt urgent CT and surgical consultation, rather than reliance on rarity or on a first-to-report claim.

## Data Availability

The original contributions presented in the study are included in the article/Supplementary Material, further inquiries can be directed to the corresponding author.
