# Supplementary material for: Case report: necrotizing fasciitis with ascending mediastinal emphysema secondary to anastomotic perforation after stapled hemorrhoidopexy
Source: Front Med (Lausanne). 2026 Oct 27;13:1914139. doi: 10.3389/fmed.2026.1914139 (PMC13750364; doi:10.3389/fmed.2026.1914139)
Supplement: Supplementary file 1 [file Table1.docx]

Supplementary Material

# 1 Supplementary Figures and Tables

**Supplementary Figure 1.** Emergency axial CT images obtained on postoperative day 3 after stapled hemorrhoidopexy (PPH), immediately before emergency surgery, with panels A–G demonstrating multidirectional gas tracking along fascial planes (arrow markers): panel A (pelvic level) shows a posterior rectal staple line defect approximately 3 cm above the dentate line with gas in the perianal and perineal tissues; panel B (ischiorectal fossa level) shows gas within the bilateral ischiorectal fossae; panel C (retroperitoneal level) shows gas tracking along the retroperitoneal fascial layers; panel D (psoas major muscle level) shows gas at the level of the psoas major muscles; panel E (subdiaphragmatic level) shows gas adjacent to the diaphragm; panel F (posterior mediastinal level) shows gas within the posterior mediastinum; panel G (cervical level) shows cranially extending gas within the mediastinal and cervical fascial spaces; panel H shows follow-up contrast-enhanced CT on postoperative day 8, revealing complete resolution of mediastinal and cervical gas with no evidence of persistent infectious spread; arrows on each image delineate the extent of subcutaneous and fascial emphysema, involved fascial layers, and necrotic areas for direct localization of imaging findings, and these features are consistent with necrotizing fasciitis spreading along contiguous fascial planes, while mediastinal and cervical gas represents radiologically detected cephalad gas tracking that has not been surgically or microbiologically confirmed as necrotizing infection.

**Supplementary Figure 2.** An anatomical flow chart adapted from the authoritative imaging literature (reference 12: Frias Vilaça A, Reis AM, Vidal IM. *The anatomical compartments and their connections as demonstrated by ectopic air*. Insights Imaging. 2013;4(6):759-772.), illustrating the propagation pathways of ectopic air through adjacent fascial layers.

| **Study (year, ref.)** | **Underlying procedure** | **Staple-line defect / perforation** | **Pelvic / perineal NF or sepsis** | **Retroperitoneal involvement** | **Mediastinal / cervical gas** | **Operative confirmation of NF compartments** | **Microbiology** | **Management** | **Outcome** |
| --- | --- | --- | --- | --- | --- | --- | --- | --- | --- |
| Cirocco 2008 (14) | Stapled hemorrhoidopexy (pooled 13 pts) | 9/13 perforation; 6 sepsis; 2 Fournier | Pelvic/perineal sepsis; Fournier gangrene in subset | Variable | Not emphasized in pooled series | Most required laparotomy + faecal diversion | Variable | Emergency laparotomy + stoma | 3 deaths (POD 4, 6, 10); others recovered |
| Pessaux 2004 (19) | PPH (44-y man) | Yes - staple-line leak on CT and at surgery | Scrotal/inguinal subcutaneous necrosis with crepitus; pelvic sepsis | Not documented as fascial NF | Gas around rectum, bladder, scrotum; not to mediastinum | Subcutaneous scrotal/inguinal necrosis debrided | Polymicrobial: E. coli, B. fragilis, Enterococcus | Debridement + end-colostomy + hyperbaric O2 | Healed by 3 mo; stoma reversed at 6 mo |
| van Wensen 2008 (20) | PPH (26-y woman) | Yes - right dorsolateral rectal perforation on DRE/laparotomy | Presacral abscess; pelvic sepsis | Presacral gas collection extending into retroperitoneum | None to mediastinum | Presacral/retroperitoneal collection; not described as fascial NF | Not stated | Laparotomy + loop ileostomy + presacral drainage | Discharged POD 19 |
| Spanish letter 2012 (7) | Longo hemorrhoidopexy (36-y woman) | No dehiscence or inflammation on CT; inferred barotrauma | None; no sepsis signs | Pneumoretroperitoneum | Massive pneumomediastinum, subcutaneous emphysema, small pneumothorax | No surgical exploration | Blood cultures negative | Conservative; imipenem/vancomycin/ceftazidime x 10 d | Discharged day 10; asymptomatic at follow-up |
| Kanellos 2004 (8) | PPH + proctoscopic dilatation (54-y man) | No staple-line defect; presumed air entry during dilatation | Perianal abscess drained | Retropneumoperitoneum | Pneumomediastinum, subcutaneous emphysema | Perianal abscess drained; no deep fascial NF | Not stated | Drainage of abscess + conservative antibiotics | Discharged day 5 |
| Ryu 2017 (21) | PPH (28-y man) | Yes - free rectal perforation POD 5 | Faecal peritonitis | Pneumoperitoneum; not described as retroperitoneal infection | None | Peritonitis; not fascial NF | Not stated | Laparoscopic lavage + primary closure, no stoma | Discharged POD 16 |
| Present case | PPH (37-y man) | Yes - 0.5 cm posterior defect, DRE-localized, directly visualized and measured at surgery | Perineal/ischiorectal NF (surgically confirmed) | Yes - retroperitoneal NF confirmed at surgery | Posterior mediastinal + cervical gas tracking only; no collection | Yes: perianal subcutaneous, superficial/deep perineal fascia, bilateral ischiorectal fossae, retroperitoneum | Negative after broad-spectrum antibiotics; interpreted cautiously | Transverse double-barrel colostomy + layered debridement + drains + imipenem/metronidazole then latamoxef | Recovered; well at 1 y |

**Supplementary Tables 1.** Comparison of the present case with the most closely related published reports of severe septic or aeriform complications after stapled hemorrhoidopexy / PPH. NF, necrotizing fasciitis; DRE, digital rectal examination; POD, postoperative day.

| **Time point** | **Clinical events and management** | **WBC (×10⁹/L)** | **CRP (mg/L)** | **Other findings** |
| --- | --- | --- | --- | --- |
| **POD 0**  **(outside hospital)** | **PPH for mixed hemorrhoids; intraoperative course uneventful** | **—** | **—** | **—** |
| **POD 1**  **(outside hospital)** | **Onset of severe anal pain disproportionate to the expected postoperative course** | **—** | **—** | **—** |
| **POD 2**  **(outside hospital)** | **High fever up to 39.3 ℃;Transient response to antipyretics; pain radiating to the lower abdomen with dyspnea** | **14.30** | **>10** | **Neutrophils 86.4%** |
| **POD 3**  **(outside hospital)** | **Recurrent fever (38.4 ℃); transfer to our hospital; perianal/gluteal swelling and erythema with palpable crepitus; digital rectal examination showing a posterior-wall depression at the staple line; contrast CT demonstrating multidirectional fascial gas without mediastinal collection (Figure 1A-G)** | **17.98** | **102.1** | **Neutrophils 89.5%; SAA >200 mg/L** |
| **POD 0**  **(emergency surgery at our hospital)** | **Emergency surgery within 12 h of diagnosis (double-barrel transverse colostomy, layered radical debridement, transanal/ischiorectal/retrorectal irrigation–drainage catheters, rubber seton counter-drainage); ICU admission; imipenem-cilastatin plus metronidazole** | **12.42** | **114.8** | **SAA >200 mg/L** |
| **POD 1**  **(our hospital)** | **Body temperature normalized; continuous irrigation and organ-supportive care continued** | **11.85** | **131.7** | **SAA 72.73 mg/L** |
| **POD 2**  **(our hospital)** | **Continuous irrigation and organ-supportive care continued; patient afebrile and hemodynamically stable; WBC and CRP trending downward; intraoperative cultures being incubated** | **10.97** | **98.4** | **Apyrexia** |
| **POD 5**  **(our hospital)** | **WBC normalized to 8.06×10⁹/L and CRP reduced >70% to 24.2 mg/L; sustained apyrexia and hemodynamic stability for >72 h; aerobic, anaerobic, and fungal cultures negative at 5 days (no organism isolated; susceptibility testing not possible); de-escalation to latamoxef monotherapy** | **8.06** | **24.2** | **CRP reduced >70% from baseline** |
| **POD 8**  **(our hospital)** | **Follow-up contrast-enhanced CT: complete absorption of mediastinal and cervical gas, no residual collection or new spread (Supplementary Figure 1H)** | **9.18** | **12.3** | **No mediastinal collection or abscess** |
| **POD 19**  **(our hospital)** | **Discharge with satisfactory wound healing and a functioning colostomy** | **—** | **—** | **—** |
| **3 months**  **(our hospital)** | **High fistula repair with seton drainage; removal of exposed staples** | **4.43** | **—** | **—** |
| **6 months**  **(our hospital)** | **V-Y advancement flap reconstruction of the perianal defect** | **8.18** | **2.8** | **—** |
| **9 months**  **(our hospital)** | **Colostomy reversal** | **3.91** | **—** | **—** |
| **12 months**  **(our hospital)** | **Normal anal function and perineal appearance; no recurrence or stenosis** | **—** | **—** | **—** |

**Supplementary Tables 2.** Clinical timeline from the index stapled hemorrhoidopexy (PPH) to the one-year follow-up, including all available inflammatory marker measurements. POD, postoperative day; WBC, white blood cell count; CRP, C-reactive protein; SAA, serum amyloid A; CT, computed tomography; vacuum sealing drainage; ICU, intensive care unit. PODs 0–3 refer to the initial PPH at the outside hospital; PODs 0–19 refer to the emergency surgery at our index hospital.
